# Where Do People Get Their Information and Contraceptives From?

**DOI:** 10.1371/journal.pmed.0020382

**Published:** 2005-10-18

There are 123 million women around the world, mostly in developing countries, who are not using contraception in spite of having expressed a desire to space or limit the numbers of their births. About 38% of all pregnancies worldwide every year are unintended, and around six out of ten such unplanned pregnancies result in an abortion. By helping women to exercise their reproductive rights, family planning programs can improve the social and economic circumstances of women and their families.

Often contraception is available only in family planning clinics and hospitals, where it is given by trained providers; however, worldwide supplies of contraceptives are available from diverse places. Studies have suggested that conventional family planning clinics might discourage some groups, such as younger people, from using their facilities, an observation backed by findings that report high awareness of contraception in some communities but low usage. But other barriers to contraceptive use include cultural issues, religion, cost, husband/partner's refusal, availability, accessibility, and fear of side effects.

In *PLoS Medicine*, Boniface A. Oye-Adeniran and colleagues say that a better understanding of families' preferences for certain distribution centers would enable providers to deliver better service. And getting contraceptives, especially condoms, closer to people in acceptable, culturally sensitive, and friendly environments could not be more urgent than now, with the need to contain the HIV/AIDS pandemic.

The team did a community-based study to examine the sources of contraceptives for users in Nigeria to identify whether there was a preference for some distribution centers. Of the 2001 respondents aged 15–49 years, 1,647 (82.3%) were sexually active, out of which only 244 (14.8%) were using contraception at the time of the study. The team found some striking trends. Most respondents got their contraceptives from chemist/patent medicine shops (19.7%), whereas only 0.8% went to family planning clinics. Married respondents preferred hospitals, health centers, and clinics—perhaps indicating their need for long-term contraception, that is, intrauterine devices and injectables. However, young unmarried respondents, who preferred condoms and pills, went to pharmacies and over-the-counter services.

The small number of respondents who got their contraceptives from family planning clinics was worrying, but showed a similar trend to Ghana, where there was also a shift from public to private sources. One factor for this development might be general staff attitude in clinics, said the authors, and the lack of youth-friendly services.

There were religious differences in usage: Catholics and Muslims preferred to use chemists/patent medicine shops for contraceptives rather than hospitals and clinics, which may have had something to do with their religions' disapproval of contraceptive use. The age of the respondent was also very important in how individuals obtained contraceptives. Most adolescents preferred chemist/patent medicine shops, but at 25 years of age and older, more people went to general or private hospitals. This behavior perhaps reflected a cultural disapproval of sex when unmarried, the group to which most of these young persons belong. The authors noted that the most popular contraceptives for younger age groups (condoms and pills) were also available over the counter. The observations in this age group are particularly relevant, said the authors, since 15–24 year olds have the greatest incidence of unwanted pregnancy and unsafe abortion.

Altogether, the study highlighted the inadequate information and counseling on the various types of contraceptive methods in Nigeria. The authors suggest that when planning contraceptive services, planners must take into account the sources of contraception identified here, and work on improving culturally relevant delivery methods, such as youth-friendly clinics.

